# A Successful Multi-Component Program for Expanding Vasectomy Services by MSI Reproductive Choices Bolivia

**DOI:** 10.12688/gatesopenres.16366.1

**Published:** 2025-10-09

**Authors:** Alison T. Hoover, Ana Cecilia Velasquez Rossi, Silvia Velasco Parihuana, Jonathan Stack, John Curington, Michel Labrecque

**Affiliations:** 1 Emory University School of Medicine, Atlanta, Georgia, 30322, USA; 2 World Vasectomy Day, New York, NY, 10011, USA; 3 MSI Reproductive Choices Bolivia, La Paz, Bolivia; 4 No-Scalpel Vasectomy International, Lutz, FL, USA; 5 Great Vas Gentle Vasectomy Center, Sarasota, FL, USA; 6 Laval University Department of Family and Emergency Medicine, Quebec City, Canada

**Keywords:** Vasectomy, provider training, male engagement, family planning costs

## Abstract

**Background:**

Vasectomy use has historically been very low in Bolivia, constituting just 0.1% of the method mix in 2021. MSI Reproductive Choices Bolivia (MSI Bolivia), one of the major reproductive health organizations in the country, sought to increase the affordability, availability, and quality of vasectomy services in their nationwide clinics and mobile units by training in-house providers to replace contracting external providers with high fees. We describe the MSI Bolivia vasectomy program in 2021 and its results over the following two years.

**Methods:**

The program included components of the Engender Health Supply-Enabling-Environment-Demand (SEED) Programing Model™ for evidence-based vasectomy programming. First, MSI Bolivia offered free vasectomies through a social media campaign during November 2021. Second, two international No-Scalpel Vasectomy (NSV) experts trained four MSI Bolivia physicians during a week-long teaching program in La Paz, Bolivia. Third, MSI Bolivia formed partnerships and held a dissemination event to publicize the campaign. MSI Bolivia continued conducting training and marketing campaigns in 2022 and 2023.

**Results:**

During the 2021 six-week promotional campaign, 884 men signed up and over 600 were scheduled for the procedure. During the training week, the trainees performed 127 supervised vasectomies. Over the following weeks, the four trained physicians performed over 300 additional unsupervised vasectomies. Two of the newly trained physicians taught NSV to seven other colleagues in 2022 and 2023. MSI Bolivia reduced the fees for a vasectomy from Bs. 1500 (USD 215) to Bs. 850 (USD 122). The number of vasectomies performed by MSI Bolivia increased from 77 in 2019 to 643, 918, and 1,135 in 2021, 2022, and 2023, respectively.

**Conclusion:**

By training their own physicians to perform NSV, reducing costs, and advertising through social media, MSI Bolivia was able to increase the availability, quality, and acceptability of vasectomy in Bolivia.

## Introduction

Bolivia, the poorest country in South America, ^1^ has nearly tripled its population in the past 50 years. ^2^ Bolivia also has a significant unmet need for contraception; only 45% of married and in-union women use any modern contraceptive methods. ^3^ The use of vasectomy for male sterilization as a permanent contraceptive method in Bolivia is very low (0.1%), particularly compared to tubal ligations for female sterilization (9.7%). ^3^ This low uptake has been attributed to a lack of contraceptive method awareness. ^4^

MSI Reproductive Choices Bolivia (MSI Bolivia), one of the major reproductive health non-governmental organizations (NGO) in the country, offers a wide range of contraception services through in-house providers and contracted external specialists. MSI Bolivia encountered difficulty providing vasectomies given the high fees charged by local specialists and low demand from patients. In-house providers performed tubal ligations, while vasectomies were contracted to external urologists. As a result, the cost of a vasectomy was almost twice that of a tubectomy. This is a stark price discrepancy as a vasectomy is customarily half the cost of a tubectomy given its speed, minimally invasive approach, and simple medical instrument and setting requirements. ^5^ In 2019, the physicians of MSI Bolivia performed 592 tubal ligations and the urologists hired by the organization performed 77 traditional scalpel vasectomies. ^6^

In November 2020, MSI Bolivia participated in World Vasectomy Day (WVD) activities for the first time ( https://wvd.org/). The initial implementation was successful, increasing the number of vasectomies provided that year to 301 and demonstrating unmet demand for the procedure. The initial experience fomented a desire among the MSI Bolivia leadership team to reduce the costs of services and improve access to quality services.

To improve its offer of male sterilization services, in 2021, in collaboration with WVD, No-Scalpel Vasectomy International (NSVI), and Laval University, Quebec, Canada, MSI Bolivia implemented a program to increase the availability, affordability, and quality of their vasectomy services. We describe the program activities and its results over the two years since its implementation.

## Methods

**Figure 1.**
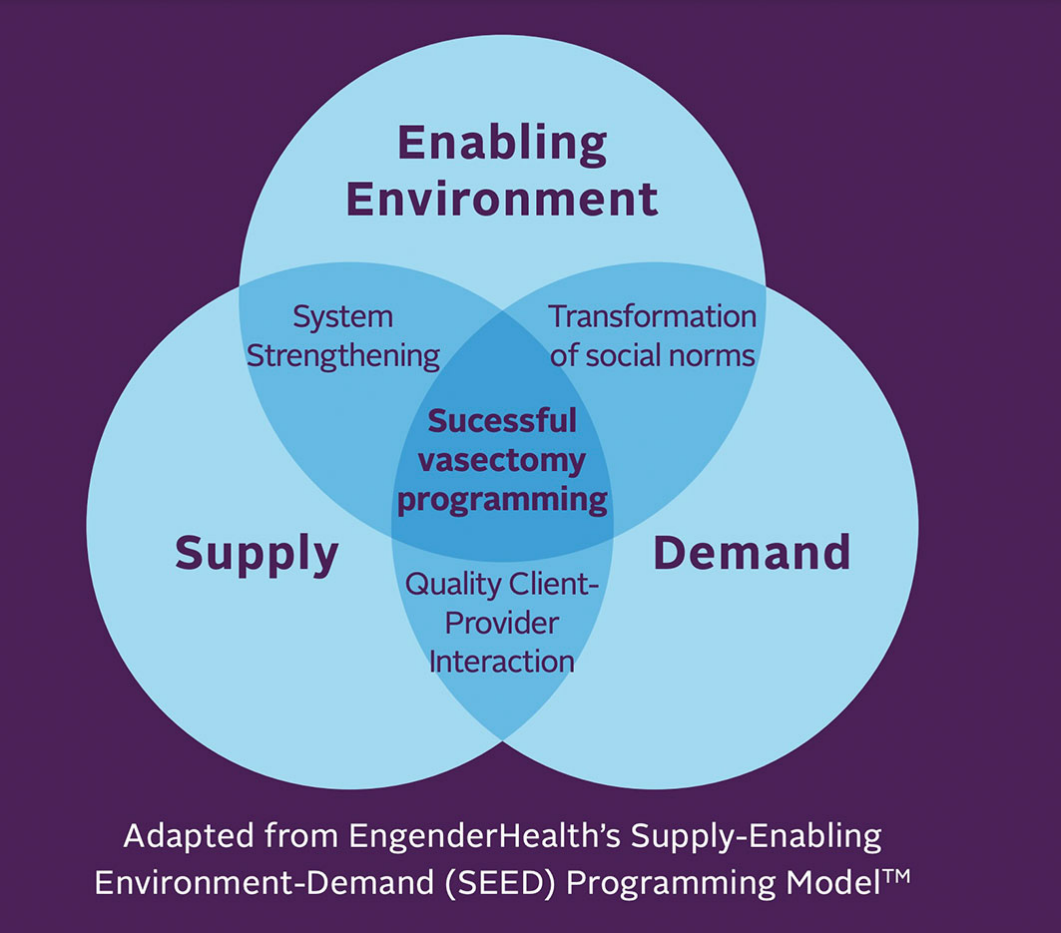
EngenderHealth Supply-Enabling-Environment-Demand (SEED) Programing Model™ adapted by FHI360 for evidence-based vasectomy programming.

The MSI Bolivia vasectomy program utilized the components of the EngenderHealth Supply-Enabling-Environment-Demand (SEED) Programing Model™, ^7^ recommended by FHI360 for promoting evidence-based vasectomy programming. ^8^ This framework identifies three main and interconnected components to create successful vasectomy program ( Figure 1). The SEED Programing Model™ activities aim at 1) generating demand in the population towards the use of vasectomy, 2) offering high-quality vasectomy services including adequate infrastructure with well-trained and motivated healthcare providers and staff, and 3) fostering an enabling environment for vasectomy within governments, communities, and civil societies.

Recognizing the role that cost was playing in limiting vasectomy access, MSI Bolivia first sought to address component 2 by training in-house providers. The goal was to transition vasectomy services in-house, reducing costs by removing the high fees charged by external contracted providers. MSI Bolivia designed a multi-phase training program that would entail the following stages:

### Initial Program Activities (July 2021-December 2021)

1. *Solidify partnerships*

MSI Bolivia partnered with WVD in July 2021 to organize a provider training. WVD identified representatives from NSVI and Laval University, authors JC and ML, to serve as master trainers. The collaborating organizations met virtually on multiple occasions in 2021 to plan the training, including identifying the number of providers to train and scheduling a week-long teaching program at the MSI Bolivia La Paz clinic from November 1 to November 6, 2021. In advance of the training, MSI Bolivia assured buy-in and support from all levels of the organization and beyond. All six clinics located in the main cities of the country (La Paz, El Alto, Sucre, Cochabamba and Santa Cruz) agreed to add no-scalpel vasectomy (NSV) in their service offering.

2. *Demand generation campaign*

The MSI Bolivia marketing team generated demand through an extensive and organized social media campaign across Facebook, Instagram, and TikTok, conducted one month in advance of the training. The social marketing campaign utilized humor ( Figure 3), factual medical information, and interviews with a Spanish-speaking physician practicing NSV advertising free availability of vasectomies during November 2021. Free-of-charge vasectomies were made possible through a grant from the Erik and Edith Bergstrom Foundation, Palo Alto, California, USA. Interested individuals were advised to contact the MSI Call Center via phone for further information and to schedule an appointment. Interested men were scheduled for a clinical consultation and evaluation.

3. *Organize provider training*

**Figure 2.**
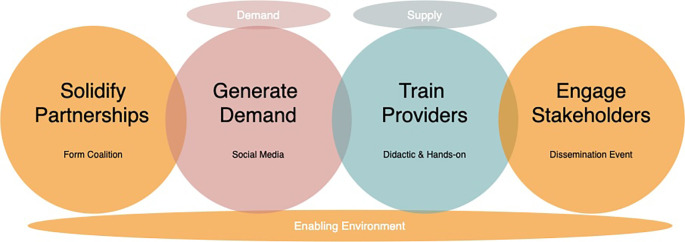
MSI Bolivia Program Model 2021 Vasectomy Campaign.

**Figure 3.**
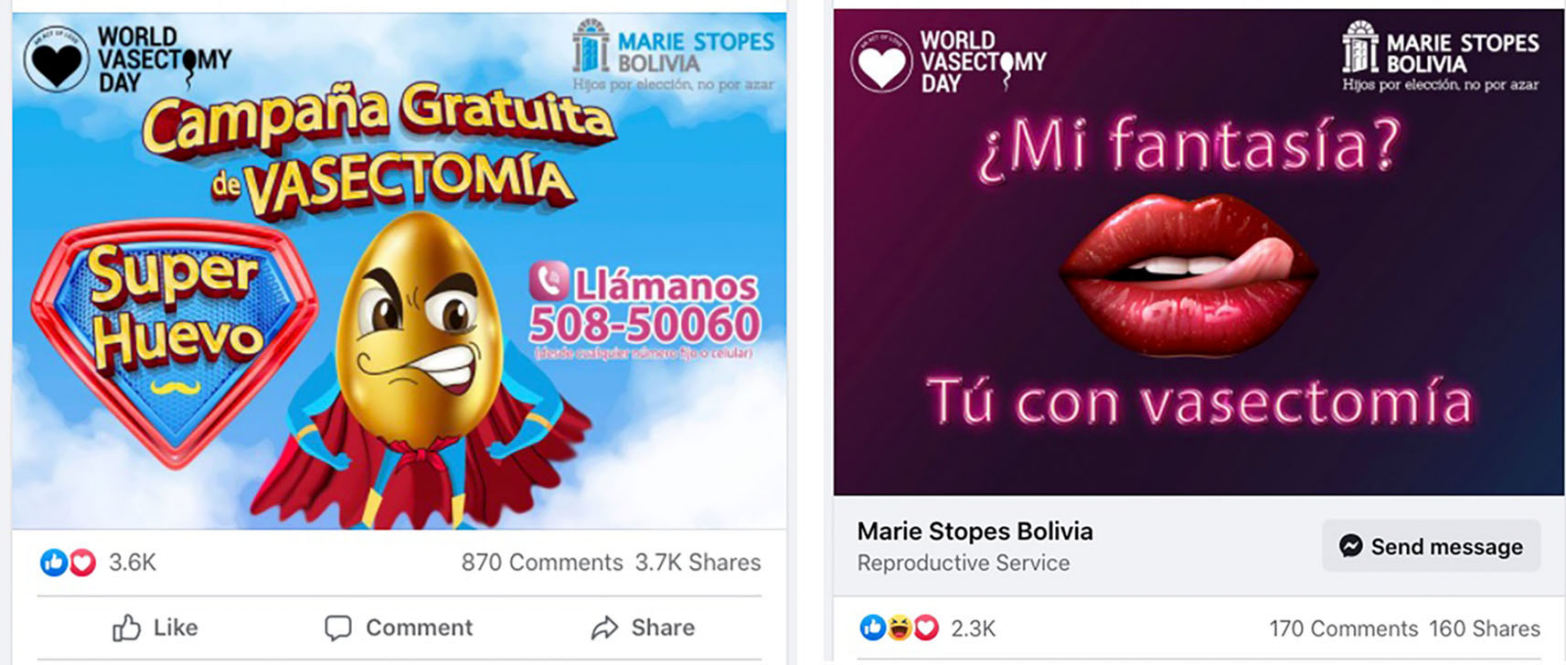
Examples of social media (Facebook) advertisements for the vasectomy campaign.

In consultations among the partner institutions, it was determined that training four providers would be feasible within the one-week supervised training period. The figure was based on the number of master trainers, the feasible number of clients, and the intention that these initial four providers would train other providers within the MSI Bolivia network. The MSI clinical team pre-selected eight candidates from across the country based on clinical skills as well as training capacity. Pre-selected candidates were invited to submit a five-minute video on how they would train colleagues to perform contraceptive implant insertion. A selection committee composed of the clinical team and the senior management team reviewed the videos, evaluating both their procedural skills and their skill in training other providers, and selected the four providers to be trained. The four physicians each represented different clinics and mobile units covering Bolivia.

MSI Bolivia led the management of key logistics, including organizing physical clinic space for procedures, procuring instrument kits, and training support staff in sterilization and procedure room turnover. Vasectomy services were provided according to MSI worldwide norms, including pre-procedure counseling and informed consent. ^9^ Days prior to the surgery, potential patients attended a vasectomy counseling session with providers trained on vasectomy counseling during which their questions were answered and eligibility confirmed. Due to the active COVID-19 pandemic at the time of the campaign, partners were not able to attend consultations or the procedure.

Upon arrival to the clinic, patients privately meet with their vasectomy provider who again reviewed the process and provided pre-op counseling, after which, the patient signed an informed consent form for the procedure. After the surgery, a physician provided post-op recommendations while he was resting in a recovery room.

**Figure 4.**
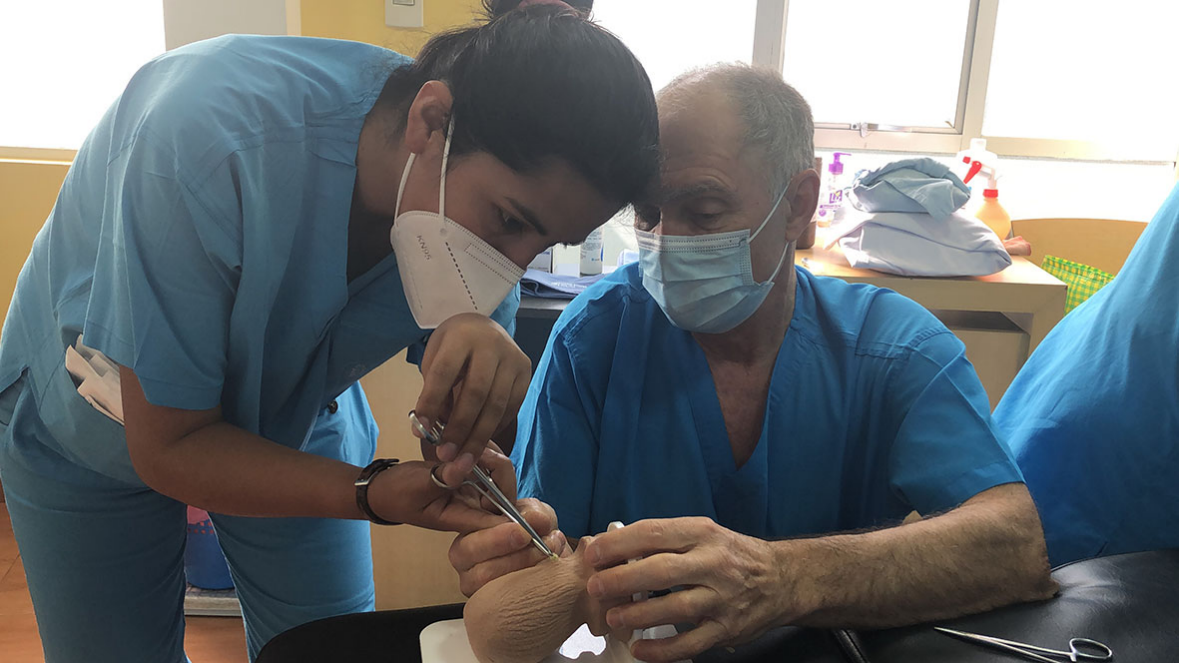
Trainee performing a simulated no-scalpel vasectomy under the supervision of her trainer.

The six-day training program involved didactic sessions, practice on vasectomy simulators prototypes (scrotal models) developed by Laval University ( Figure 4), and supervised practice on humans. The techniques taught were the mini-needle technique to perform local anesthesia, ^10^ the NSV to isolate and expose the vas deferens, ^11^ and thermal cautery and fascial interposition to occlude the vas, ^12^ as recommended in international vasectomy guidelines. ^13^

For each vasectomy performed, the surgical team consisted of one master trainer and two trainees on a rotating schedule to ensure each trainee learned alongside both master trainers and each of their peers. One trainee would perform the vasectomy while the trainer and the other trainee observed. The trainees were evaluated daily using detailed written spreadsheets (see Appendix 1). These spreadsheets had columns for each step of the procedure, allowing the trainers to focus on the specific details needed to advance the skills of the trainees. Both the trainer and trainee used the same tool to evaluate the trainee performing the surgery, as MSI Bolivia planned that trainees would instruct their peers in the future.

The six-day training program began with one half-day of watching presentations and practicing on vasectomy simulators (scrotal models) followed by three supervised vasectomies on patients by each trainee. The five subsequent days included a one-hour session at the beginning of the day devoted to reviewing and demonstrating acquired skills on scrotal models followed by six supervised vasectomies. At the end of each training day, trainers and trainees met for 30- to 60-minute debriefing sessions. They reviewed new skills practiced during the day. They discussed what went well and what to improve based on the evaluation forms. They also planned specific skills to practice over the next days. Over the six days, each trainee completed 33 supervised cases. To support continued learning, trainees continued to work in pairs for their first 100 procedures.

4. *Engage local stakeholders*

MSI Bolivia held a press conference and informational event called “VasectoMITOS” (vasecto-myths, in English) to publicize the campaign, engage stakeholders, and disseminate learnings during the training week. Attendees included sexual and reproductive health advocates and organizations in Bolivia, as well as the contracted urologists already working with the organization and other vasectomy providers. The event included a panel presentation, a conversation with a vasectomy patient, promotional videos, and a reception. Other efforts to engage stakeholders included inviting family planning organizations, specialists, and urologists to observe the training at the La Paz clinic to foster cooperation.

### Follow-up Program Activities (January 2022-December 2023)

Once a sufficient cadre of providers had been trained, starting in May 2022, MSI Bolivia reduced the fees charged for a vasectomy from 1500 Bolivianos (USD 215) to 850 Bolivianos (USD 122) in all their clinics nationwide. In line with World Vasectomy Day activities in November 2022, MSI Bolivia conducted another free vasectomy campaign, advertised via social media through November and half of December 2022. Similarly, in 2023, MSI Bolivia organized another campaign, this time from October through November 2023. Both campaigns were held in the cities of La Paz, El Alto, Cochabamba, Santa Cruz de la Sierra, and Sucre.

Two of the newly trained physicians in November 2021 taught NSV to seven other MSI Bolivia colleagues in 2022, and an additional four colleagues in 2023, replicating the training techniques from the original training. Trainees practiced on scrotal models, performed vasectomies under supervision, debriefed their training progress daily and were left to work in pairs supporting each other immediately after the training.

## Results

### Initial Program Activities (July 2021-December 2021)

During the 2021 social media campaign, MSI Bolivia received triple the number of calls requesting appointments compared to the average number of calls received each month. A total of 884 men registered, 703 attended an initial consultation, and over 600 were scheduled for the procedure.

The social media campaign generated tens of thousands of views. The initial advertising post on Facebook had 3,600 reactions, 870 comments, and 3,700 shares ( Figure 2), while another video on TikTok generated 86,400 views and 7,973 reactions.

One hundred and thirty-three (133) men were scheduled for a vasectomy during the November 1-6, 2021 training week. Five did not present for their appointment, leading to a total of 127 vasectomies performed. During the first 2.5 days, the trainers administered the local anesthesia, and maintained a low threshold for taking over the case if the trainees faltered. According to the evaluation tool, the trainees reported increasing confidence and skill each day in anesthesia, isolation of the vas using no-scalpel technique, mucosal thermal cautery, and fascial interposition. After day four, the trainers only provided remote supervision with the two trainees working together and supervising each other. All four doctors exhibited an ability to conduct vasectomies independently with various skill levels by the end of the week. No patients suffered complications, either during the procedure or in the first days following the surgery.

Trainee feedback was overall very positive. They rated the presentations, the rotational training approach, and the practicing with vasectomy simulators very highly. Over the following weeks, the four trainees performed over 300 additional unsupervised vasectomies in La Paz, Cochabamba, and Santa Cruz de la Sierra. During this period, they worked in pairs for mutual support. The vasectomy expert trainers continued to provide support to trainees through both WhatsApp and email.

The “VasectoMITOS” event held during the training week gathered to 40 participants from various organizations and was reported in the local media. The event attendees included urologists from around Bolivia, representatives from ProMujer, CIES, Plan International, CECI, InnovaSalud, Servicio Plurinacional de la Mujer (a government agency that is part of the Ministry of Justice), Organon, UNFPA Bolivia, SEDES La Paz (state health department), and Sinergía.

### Follow-up Program Activities (2022-2023)

During 2022, MSI Bolivia went from four to 11 trained providers country wide. In April 2022, two of the initially trained physicians taught NSV to two colleagues from La Paz offering mobile services to remote areas served by the La Paz and El Alto clinics. They subsequently trained two others from Cochabamba in August 2022, two more in November 2022 (one from La Paz and one from Sucre) and a final one in Santa Cruz in December 2022.

MSI Bolivia received 4,926 and 6,688 calls, and scheduled 803 and 1,390 consultations and 540 and 776 procedures during the 2022 campaign from November to mid-December and the 2023 campaign from October to November, respectively. Table 1 presents the number of vasectomies performed annually in each of the MSI Bolivia clinics from 2019 to 2023. The number of vasectomies had already increased in 2020 before availability of in-house vasectomy providers. However, compared to 2020, the number doubled in 2021, tripled in 2022, and almost quadrupled in 2023. MSI Bolivia’s own physicians performed 66% (431/643), 94% (864/918), and 100% (1135/1135) of the vasectomies provided by the organization in 2021, 2022, and 2023, respectively, Vasectomy was performed in all six of their clinics.

**Table 1.** Number of vasectomies performed in MSI Bolivia clinics, 2019-2023.

Clinic location	Number of vasectomies performed				
	2019	2020	2021	2022	2023
La Paz	29	104	293	211	379
El Alto	0	36	0	210	237
Cochabamba	38	97	197	352	274
Sucre	0	13	19	49	49
Santa Cruz	10	51	134	94	150
Tarija	0	0	0	2	46
Total	77	301	643	918	1135

**Table 2.** Vasectomies performed by MSI Bolivia providers as of 2023 by clinical practice location, training month and year, and trainer.

Vasectomy provider	Clinical practice location	Month and year of training	Trainer	Number of vasectomies performed by the end of 2023 *
#1	La Paz	Nov-21	WVD	519
#2	El Alto	Nov-21	WVD	263
#3	Sucre/Santa Cruz	Nov-21	WVD	459
#4	Tarija	Nov-21	WVD	104
#5	La Paz Mobile Unit	Apr-22	#2	56
#6	El Alto Mobile Unit	Apr-22	#1	106
#7	Cochabamba	Aug-22	#2	146
#8	Cochabamba	Aug-22	#1	206
#9	La Paz	Nov-22	#2	235
#10	Sucre	Nov-22	#2	96
#11	Santa Cruz	Dec-22	#2	113
**Total**				**2,303**

* These numbers exclude the 127 vasectomies performed during the initial training by WVD in November 2021.

The 11 MSI Bolivia-trained physicians performed 2,303 vasectomies by the end of 2023. Most physicians, even the most recently trained, performed more than 100 procedures after their training. It was initially difficult to stimulate demand in remote areas served by mobile units (21 vasectomies in 2022) due to resistant cultural norms around family planning uptake generally and machismo attitudes surrounding vasectomy in particular. Targeted peri-urban campaigns in 2023 helped improve access and overcome some of the resistance, with 141 vasectomies performed in 2023.

**Figure 5.**
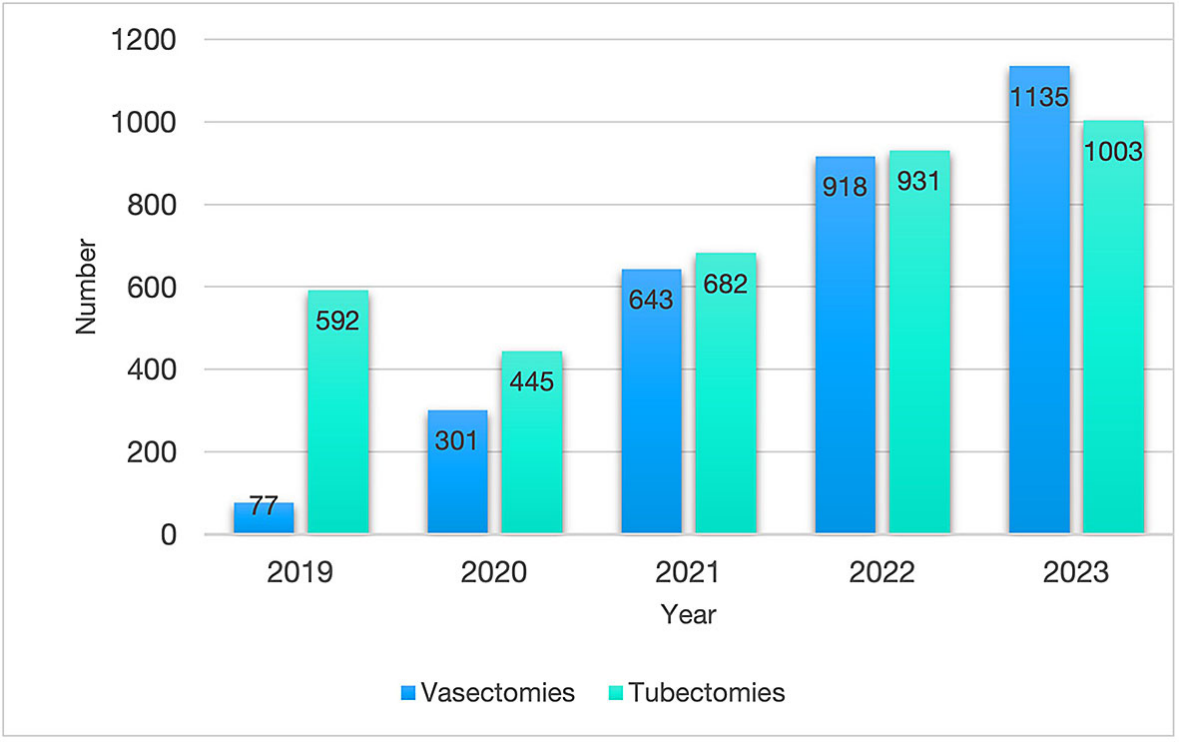
Number of vasectomies and tubectomies performed in the MSI Bolivia vasectomy clinics from 2019 to 2023.

Although both the number of vasectomies and tubectomies performed in the MSI Bolivia clinics increased between 2019 and 2023, the ratio of tubal ligations to vasectomies changed during this period ( Figure 5). From 7.1 tubectomies to one vasectomy in 2019, this ratio decreased to 1.5 in 2020 and to 0.9 in 2023, indicating that more vasectomies than tubal ligations were performed in MSI Bolivia clinics in 2023.

## Discussion

By training their own physicians to perform NSV, reducing the costs, and stimulating demand through social media, MSI Bolivia was able to increase the availability, affordability, and acceptability of vasectomy in Bolivia.

The timing for restructuring and increasing MSI Bolivia vasectomy services proved to be excellent. A number of key components of the SEED Programming Model ^TM^ were already in place within the organization. MSI Bolivia had administrative, financial and management systems in addition to clinical infrastructure in place to initiate and sustain a high-quality vasectomy program. MSI Bolivia was already offering limited vasectomy services governed by MSI Contraceptive Choices international standards of practice. ^9^ MSI Bolivia had a well-established reputation for high-quality reproductive health services, including a robust social media following. Social media was an effective outreach strategy in Bolivia, with over 95.3% of the Bolivian population over 13 years of age reporting using at least one social media platform. ^14^ Moreover, the high number of men responding to the campaign advertisements demonstrated significant unmet demand for vasectomy services among the Bolivian population. Centrally, training young, motived, and skilled in-house physicians already performing other contraceptive surgical procedures was one of the greatest contributors to the improvement of vasectomy services at MSI Bolivia and the sustained and increasing demand since the training. The intentional selection process contributed greatly to the success of the program.

Coalescing expert partner organizations brought expertise in demand generation through partnership with WVD and in the organization and logistics of the training activities through partnership with NSVI and Laval University. After the six-day training workshop, combining simulated practice on scrotal models and one-to-one stepwise supervision of vasectomy cases, all trainees mastered evidence-based surgical techniques. Moreover, they were able to replicate their apprenticeship with their colleagues over the following year. This achievement strengthened the system already in place, stimulated motivation at all levels of the organization, improved the quality of client-provider interactions and, backed by the social media campaigns, fostered social norm transformation. ^8^

The success of MSI Bolivia inspired other Bolivian NGOs and the Bolivia Ministry of Health to enhance their vasectomy services. From September to November 2023, led by WVD with support of United Nations Population Fund (UNFPA) and in collaboration with No-Scalpel Vasectomy and Laval University, physicians from ProSalud, CIES and the Ministry of Health hosted a similarly structured training. One of the four MSI Bolivia physicians trained in November 2021 served as a trainer. In total, one physician from CIES, two physicians from ProSalud (who, with the support of two urologists, subsequently trained four additional colleagues: two from Cochabamba, one from Oruro, and one from La Paz), one physician from an academic institution, and four physicians from the Ministry of Health were trained. Similarly to MSI Bolivia, ProSalud began offering vasectomies in-house for the first time. These expanded public-private partnerships and provider trainings are evidence of national interest, enthusiasm, and collaboration toward increasing access to and the quality of vasectomy services in the country.

The prevalence of vasectomy as a contraceptive method in Bolivia among women 15-49 years old is currently estimated at 0.1%. ^3^ According to an analysis by Jacobstein et al., if about 3,000 vasectomies were performed each year in the country, the prevalence could raise to 0.5% in 5 years and to about 1% in 10 years. ^14^ Considering the steady increase in number of vasectomies performed in MSI Bolivia clinics, and the strong interest among other NGOs including CIES and ProSalud and the Ministry of Health in offering NSV services, this number may be rapidly exceeded. Bolivia could replicate the growing prevalence of use of vasectomy observed in recent years in some Latin America countries, namely Costa Rica, Colombia, and Mexico. ^15^

There are still barriers remaining to the widespread use of vasectomy in Bolivia. The MSI Bolivia program may not be generalizable in other settings. Not all the organizations with interest in offering NSV services in Bolivia have the same leadership, infrastructure, and financial and human resources required to implement and sustain a vasectomy program. Continuous support from organizations such as WVD, UNFPA, and other funding agencies may be crucial for sustaining early successes.

In many low-resource countries, lack of knowledge and negatives attitudes about vasectomy are cited as important factors limiting men and couples to choose male sterilization as a contraceptive option. ^16^ This is probably still the case in Bolivia, as evidenced through the limited uptake of family planning generally and vasectomy specifically in rural areas with mobile outreach units. Access to minimally invasive NSV that is more appealing to men than standard vasectomy is very recent and limited in the country. A comprehensive approach involving improved access to contraceptive services, awareness promotion activities, and supportive policies will be needed for long-term and pervasive social norm transformation, particularly in rural areas.

In conclusion, the MSI Bolivia vasectomy program based on the SEED Programing Model™ within a network of in-house NSV trained providers was very successful in increasing vasectomy use in the three years since its implementation. It inspired other Bolivian governmental and nongovernmental organizations to follow their tracks. Continuous monitoring of vasectomy services at MSI Bolivia and in Bolivia in general will valuable. Many countries and organizations struggling to increase the use of vasectomy in their setting will likely benefit from the experience and success of MSI Bolivia.

## Ethics and consent

This study describes a training program and does not include research participants. All individuals receiving a vasectomy signed an informed consent form, but only in relation to their medical procedure, which is not the focus of this paper.

## Data Availability

Data described in this manuscript are observational in nature or derived from clinic electronic medical records pulled by clinicians with preexisting access to the data. The data analyzed in this study are not publicly available as they are sensitive patient information and are subject to privacy and confidentiality laws. Minimal de-identified aggregate data may be made available upon reasonable request and in tandem with Institutional Review Board approval.

## References

[1] World Population Review: Poorest Countries in South America 2024.Accessed January 5, 2024. Reference Source

[2] World Bank: World Bank Open Data - Bolivia.Accessed January 5, 2024. Reference Source

[3] United Nations Department of Economic and Social Affairs, Population Division. World Contraceptive Use 2022. 2022. Reference Source

[4] Kent MM : *Bolivia, Slow Fertility Decline and Some Improvements in Health Indicators.* Ministerio de Salud y Deportes (MSD), Programa Reforma de Salud (PRS), Instituto Nacional de Estadística (INE) y Macro International. October 2009. *Encuesta Nacional de Demografía y Salud ENDSA*2008. La Paz, Bolivia: MSD, PRS, INE y Macro International. Accessed September 26, 2025. Reference Source

[5] KFF: *Sterilization as a Family Planning Method.* KFF;December 14, 2018. Accessed January 5, 2024. Reference Source

[6] MSI Reproductive Choices: Country Management Reporting Records.

[7] EngenderHealth: *The SEED assessment guide for family planning programming.* New York:2011. Reference Source

[8] Packer C Perry B Chin-Quee D : How to Create Successful Vasectomy Programs. 2016. Accessed January 5, 2024. Reference Source

[9] Marie Stopes International: Guidelines for Vasectomy v2.0. September 2020.

[10] Shih G Njoya M Lessard M : Minimizing pain during vasectomy: the mini-needle anesthetic technique. *J. Urol.* 2010;183(5):1959–1963. 10.1016/j.juro.2010.01.006 20303536

[11] Li SQ Goldstein M Zhu J : The no-scalpel vasectomy. *J. Urol.* 1991;145(2):341–344. 10.1016/s0022-5347(17)38334-9 1988727

[12] Labrecque M : Vasectomy occlusion technique combining thermal cautery and fascial interposition. *Int. Braz. J. Urol.* 2011;37(5):630–635. 10.1590/s1677-55382011000500010 22099275

[13] Labrecque M : Are evidence-based vasectomy surgical techniques performed in low-resource countries? *Gates Open Res.* 2019;3:1462. 10.12688/gatesopenres.12986.2 31259316 PMC6584738

[14] Kepios: *Digital 2022: Bolivia.* 2022. Accessed November 1, 2023. Reference Source

[15] Jacobstein R Radloff S Khan F : Down But Not Out: Vasectomy Is Faring Poorly Almost Everywhere-We Can Do Better To Make It A True Method Option. *Glob. Health Sci. Pract.* 2023;11(1):e2200369. 10.9745/GHSP-D-22-00369 36853640 PMC9972380

[16] Shattuck D Perry B Packer C : A Review of 10 Years of Vasectomy Programming and Research in Low-Resource Settings. *Glob. Health Sci. Pract.* 2016;4(4):647–660. 10.9745/GHSP-D-16-00235 28031302 PMC5199180

